# The LAT1 inhibitor JPH203 reduces growth of thyroid carcinoma in a fully immunocompetent mouse model

**DOI:** 10.1186/s13046-018-0907-z

**Published:** 2018-09-21

**Authors:** Pascal Häfliger, Julien Graff, Matthias Rubin, Amandine Stooss, Matthias S. Dettmer, Karl-Heinz Altmann, Jürg Gertsch, Roch-Philippe Charles

**Affiliations:** 1 Institute of Biochemistry and Molecular Medicine, and Swiss National Center of Competence in Research (NCCR) TransCure, University of Bern, Bühlstrasse 28, CH-3012 Bern, Switzerland; 2 Institute of Pharmaceutical Sciences, and Swiss National Center of Competence in Research (NCCR) TransCure, ETH Zürich, Vladimir-Prelog-Weg 4, CH-8093 Zürich, Switzerland; 3 Institute of Pathology, University of Bern, Murtenstrasse 31, CH-3008 Bern, Switzerland; 4 Present address: Herbert Irving Comprehensive Cancer Center, Columbia University Medical Center, New York, USA

**Keywords:** Genetically engineered mice, ATC, BRAF, *SLC7A5*, mTOR

## Abstract

**Background:**

The L-type amino acid transporter 1 (LAT1/*SLC7A5*) transports essential amino acids across the plasma membrane. While LAT1 is overexpressed in a variety of human neoplasms, its expression and its role in thyroid cancer is currently unknown. Anaplastic thyroid carcinoma (ATC) is a highly aggressive malignancy for which no effective therapy exists. The purpose of this study was to explore whether the inhibition of LAT1 in ATC would affect tumor growth both in vitro and in vivo.

**Methods:**

LAT1 was pharmacologically blocked by JPH203 in human ATC and papillary thyroid cancer (PTC) cell lines. The effects on proliferation and mTORC1 activity were addressed in vitro. A genetically engineered mouse model of ATC was used to address the effect of blocking LAT1 on tumor growth in vivo. *SLC7A5* transcription was measured in patient-derived ATC samples to address the clinical relevance of the findings.

**Results:**

LAT1 block by JPH203 reduced proliferation and mTORC1 signaling in human thyroid cancer cell lines. *SLC7A5* transcription was upregulated in ATC tissues derived from a genetically engineered mouse model and in ATC samples recovered from patients. JPH203 treatment induced thyroid tumor growth arrest in vivo in a fully immunocompetent mouse model of thyroid cancer. Additionally, analysis of publicly available datasets of thyroid carcinomas revealed that high LAT1 expression is associated with potentially untreatable PTC presenting reduced NIS/*SLC5A5* transcription and with ATC.

**Conclusions:**

These preclinical results show that LAT1 inhibition is a novel therapeutic approach in the context of thyroid cancers, and more interestingly in untreatable thyroid cancers.

**Electronic supplementary material:**

The online version of this article (10.1186/s13046-018-0907-z) contains supplementary material, which is available to authorized users.

## Background

A major hallmark of cancer is sustained proliferative signaling [1], which allows cells to divide rapidly in an uncontrolled way. Proliferation is highly energy demanding and depends on the availability of nutrients in the tumor microenvironment. Among these nutrients, essential amino acids (EAA) require an active transport across the plasma membrane in order to be available for protein synthesis. The L-type amino acid transporter 1 (LAT1/*SLC7A5*) is a membrane transporter for EAA such as leucine and phenylalanine. LAT1 functions in a Na^+^-independent manner [2] and exchanges glutamine for substrate EAA [3]. LAT1 is covalently attached to 4F2hc (also termed CD98 or *SLC3A2*), which is required for its trafficking to the membrane [4]. LAT1 is expressed in the brain endothelium, placenta and spleen [2, 5, 6]. Interestingly, LAT1 has been shown to be overexpressed during the development of a variety of different tumors such as prostate, breast, gastric, lung and pancreatic cancers [7–11]. It works in concert with ASCT2/*SLC1A5* that effects inwards transport of glutamine and thus participates in establishing the glutamine gradient that is required for LAT1-dependent leucine uptake [3]. Likewise, ASCT2/*SLC1A5* is also elevated in numerous cancers [12–14].

Thyroid cancer is the most frequent endocrine neoplasia and its incidence has dramatically increased during the past 30 years [15]. In 2016, thyroid cancer ranks fifth in the number of all estimated new cases of cancer in females in the US [16]. Papillary thyroid carcinoma (PTC) is the most frequent type, representing about 80% of all cases [17]. PTC is well managed in the clinic by thyroidectomy alone or complemented with radioactive iodine treatment with a 5-years survival rate of 94% [18]. In contrast, patients that are diagnosed with anaplastic thyroid carcinoma (ATC), one of the most aggressive tumors known in humans, have a dismal prognosis with a median survival rate of 5 months and a 20% 1-year survival [19]. Although ATC represents only about 2% of thyroid carcinoma [17], its invariable fatal outcome underscores that new therapeutic strategies are urgently needed to combat this highly aggressive disease.

BRAF is part of the RAS-RAF-MEK-ERK pathway, and is mutated in human cancers with a frequency of 8% [20]. More than 90% of *BRAF* mutations are caused by a T1799A transversion, resulting in BRAF^V600E^ mutant protein expression. In thyroid cancer, this mutation is found in 40% of the cases overall and 25% of ATC cases [21]. A conditional mouse model mimicking BRAF^V600E^ is able to initiate tumor formation in lung [22], melanocytes [23] and pancreas [24]. Regarding thyroid cancer, thyroid-specific BRAF^V600E^ expression leads to PTC in mice, thus confirming the importance of the mutation in this pathology [25]. Phosphoinositol 3 kinase (Pi3’K) mutations are frequent (23%) in ATC [26]. When the activated mutant PIK3CA^H1047R^ is expressed in mice thyroids concomitantly with BRAF^V600E^, the progression from PTC to ATC resulting in mice death can occur within 3 to 6 months [27]. This model closely mimics the human ATC.

Despite previous associations between LAT1 and other cancer types, the role of LAT1 in thyroid cancer has not yet been studied. Here, we assessed whether LAT1 is upregulated during the progression of thyroid cancer and whether the inhibition of LAT1 by a potent and selective LAT1 inhibitor would reduce thyroid cancer growth both in vitro and in vivo. Our data show that LAT1 is highly expressed in human thyroid cancer cell lines and that LAT1 inhibition by JPH203 reduces proliferation and impairs mTORC1 activity. In vivo, JPH203 exerted a stalemate of tumor burden increase in a mouse model of thyroid cancer. In line with these findings, *SLC7A5* transcription was elevated in human PTC samples and even further increased in ATC samples compared to normal thyroid tissues. In PTC, *SLC7A5* level was found associated with worse prognosis and reduced *SLC5A5* (Sodium iodine symporter) transcription. This is the first study showing a critical role for LAT1 in a solid tumor in a genetically engineered mouse model baring fully competent immune system. LAT1 inhibition in highly aggressive thyroid cancers might be a novel therapeutic strategy to stop tumor growth in thyroid cancer patients.

## Methods

### JPH203

JPH203 was synthesized following the route described in ref. [28]. The analytical data for all intermediates and JPH203 (^1^H-NNMR, ^13^C-NMR, ^18^F-NMR, where applicable) were in agreement with the expected structures. The analytical data for JPH203 are included in the SI.

### Animals

All animal experiments were in accordance with the Swiss animal welfare law and were approved by the local veterinary authority in Bern (license number: BE92/15). *Braf*^*CA*^, *Pik3ca*^*Lat*^ and *Thyro::Cre*^*ERT2*^ mice were described previously [22, 25, 27, 29]. Thyrocyte-specific Cre^ERT2^ activation was achieved by intraperitoneal injection of 1 mg of tamoxifen dissolved in 100 μl of peanut oil into 5–7 weeks old mice for five consecutive days.

### MEK/PI3K inhibition in mice

Double mutant BRAF^V600E^ PIK3CA^H1047R^ mice, two months after tamoxifen injections were treated by oral gavage with 5 mg/kg of PD-325901 or 50 mg/kg of GDC-0941, formulated in Hydroxypropyl methylcellulose 0.5%, Tween-80 0.2% for 10 consecutive days. Vehicle only was used as a control. Tumours were resected 4 h after the last gavage and snap frozen prior to RNA extraction. Small drug inhibitors were purchased from Abmole.

### Cell lines

8505c cells were purchased from ECACC (cat. no. 94090184), LNCaP cells were purchased from Sigma-Aldrich (cat. no. 89110211), SW1736 cells were purchased from CLS (cat. no. 300453). Hth104, KTC1 and TPC-1 cells were a gift from Prof. James Fagin (MSKCC, New York, USA) and were cultured in RPMI medium (Sigma-Aldrich, cat. no. R8758). K1 cells were a gift from Prof. James Fagin (MSKCC, New York, USA) and cultured in DMEM:F12 medium (Gibco, cat. no. 11320–074). HT-29 cells were purchased from ATCC (cat. no. HTB-38) and cultured in Modified McCoy’s 5A medium (Gibco, cat. no. 26600–080). Media of all cell lines were supplemented with 10% FBS (Gibco, cat. no.10270), 100 U/ml penicillin (Sigma-Aldrich, cat. no. P0781), 0.1 mg/ml streptomycin (Sigma-Aldrich, cat. no. P0781). Media of 8505c, LNCaP and K1 cells were additionally supplemented with 1X Non-Essential Amino Acids (NEAA) (BioConcept, cat. no. 5-13 K00). All cell lines were kept up to 50 passages or 6 months, whichever limit was reached first.

### Patients

The use of PTC and ATC patient histological tissue sections obtained at the University Hospital of Bern (Inselspital) was approved by the Cantonal Ethics Committee Bern (ref. no. 200/2014). Histological diagnosis was performed by one of the authors (M.S. D.).

### Real-time PCR

Total RNA from cells and mouse thyroid tissue was extracted by using Qiazol reagent (Qiagen, cat. no. 79306) according to the manufacturer’s protocol. Total RNA from formalin-fixed paraffin-embedded human thyroid cancer tissue was extracted using a High Pure FFPET RNA isolation kit (Roche, cat. no. 06868517001) according to the manufacturer’s protocol. Reverse-transcription was performed with Superscript II (Invitrogen, cat. no. 18064–014) following the manufacturer’s protocol. TaqMan real-time primer/probe sets were purchased from Applied Biosystems: *Slc7a5* Mm00441516_m1, *SLC7A5* Hs00185826_m1, *Slc3a2* Mm00500521_m1, *Slc1a5* Mm00436603_m1, *Slc38a2* Mm00549967_m1, *Atf5* Mm04179654_m1, *actb* 4352341E, *Tuba1a* Mm00846967_g1, *ACTB* 4326315E. Quantifications were performed using a ViiA 7 real-time PCR machine (Applied Biosystems). Relative differences in gene expression levels were assessed by normalizing the Ct value of the target gene to the Ct value of the housekeeping gene and quantified by the 2^-∆∆Ct^ method.

### Western blotting

Proteins were extracted in RIPA buffer (20 mM Tris-base pH 8, 150 mM NaCl, 1% Triton X-100, 0.1% SDS, 0.5% sodium deoxycholate) supplemented with Halt protease/phosphatase inhibitor cocktail (Pierce, cat. no. 78444). Protein concentration was assessed by Pierce BCA protein assay kit (Thermo Scientific, cat. no. 23225) and 10–30 μg total protein were separated by SDS-PAGE. Protein gels were transferred onto nitrocellulose membranes and blocked in 5% BSA in TBS. Primary antibody used (purchased from Cell signaling unless specified): P-p70S6K (cat. no. 2708), p70S6K (cat. no. 2708) P-S6 S240/244 (cat. no. 5364), P-S6 S235/236 (cat. no. 4858), S6 (cat. no. 2217), P-ERK (cat. no. 9107), Tot-ERK (cat. no. 4370), P-AKT S473 (cat. no. 4060), pan-AKT (cat. no. 2920), β-actin (Sigma-Aldrich, cat. no. A5316) and LAT1 (TransGenic Inc., cat. no. KE026). Primary antibodies were detected by using goat anti-rabbit IR680 (Li-Cor Bioscience, cat. no. 926–68071) and goat anti-mouse IR800 (Li-Cor Bioscience, cat. no. 926–32210) and imaged by a LI-COR OdysseySA imaging system.

### Proliferation assays

Cells were seeded at 10% confluency in a 96-well plate. They were allowed to adhere overnight, washed once with PBS and incubated in custom media: Earle’s balanced salt solution (EBSS) plus 5.5 mM D-glucose, 0.025 mM phenol red (Gibco, cat. no. 24010–043), 10% FBS, 100 U/ml penicillin, 0.1 mg/ml streptomycin, 2 mM L-glutamine, 1X NEAA (BioConcept, cat. no. 5-13 K00) and 1X MEM vitamin solution (Gibco, cat. no. 11120–052). For proliferation assays at different concentrations of essential amino acids (EAA), medium described above was supplemented with 1X MEM amino acids solution (Gibco, cat. no. 11130–051; see Additional file 1: Table S1 for detailed composition) and a two-fold serial dilution with the EAA-free medium was performed. Dose-response curves were carried out in custom medium with 0.125X MEM amino acids solution (Table S1). LNCaP wells were coated overnight with 0.1 mg/ml poly-D-lysine (Sigma-Aldrich, cat. no. P6407-5MG) in order to avoid detachment during washings. Cells were treated with the indicated concentration of JPH203 for two population doubling times (48 h for 8505c, SW1736 and K1; 96 h for LNCaP) and fixed with 10% buffered formalin, stained with 0.2% crystal violet (Sigma-Aldrich, cat. no. C3886-25G) in 2% ethanol, washed 5 times in dH_2_O and lysed with 100 μL 1% SDS. OD at 550 nm was measured with a microplate reader and normalized to DMSO control.

### Ultrasound imaging and JPH203 treatment in vivo

Tumor burden was measured by quantifying the area of the largest thyroid cross-section observed (mm^2^) and normalized to the size at the beginning of the experiment as described previously [30]. JPH203 was diluted in 40% (*w*/*v*) Captisol® (CyDex Pharmaceuticals) at a concentration of 7.5 mg/ml and sonicated for 1 min. Mice were treated for 5 consecutive days/week for 45 days with 200 μl of 7.5 mg/ml JPH203 or vehicle by i.p. injection, corresponding to a dose of 50 mg/kg/day.

### Immunofluorescence staining and TUNEL

Tissue section and Tunel stains were performed as described before [30]. Primary antibodies anti-Ki67 (Abcam cat. no. ab16667,) and anti-BrdU (Abcam cat. no. ab6326,) were incubated overnight at 4 °C. Secondary antibodies goat anti-rabbit Alexa-488 (Invitrogen, cat. no. A11034,) and goat anti-rat Alexa-555 (Invitrogen, cat. no. A21434,) were used to detect antigen-antibody complexes; slides were counter-stained with DAPI (500 ng/ml, Sigma Aldrich, cat. no. 32670-25MG).

### Slides scanning

Slides were scanned using a Pannoramic Midi digital slide scanner (3DHISTECH Ltd.) and analyzed by CellQuant software (3DHISTECH Ltd.). To quantify Ki67/BrdU positive cells, an average of 8 nonconsecutive sections of the tumor were used for each sample, 4 sections for TUNEL.

### ^3^H-leucine uptake assay

Cells were seeded at 60% confluency in a 96-well plate using complete culture medium and cultured until confluent. Cells were washed three times with 37 °C pre-warmed Na^+^-free Hank’s balanced salt solution (HBBS) containing 125 mM choline-Cl, 25 mM HEPES, 4.8 mM KCl, 1.2 mM MgSO_4_, 1.2 mM KH_2_PO_4_, 1.3 mM CaCl_2_ and 5.6 mM glucose (pH 7.4) and further incubated in the same buffer at 37 °C for 7 min. L-leucine uptake was measured for 3 min at 37 °C in the same buffer containing 30 μM L-[^3^H]leucine (60 Ci/mmol) and different concentrations of JPH203. Uptake was terminated by removing the solution followed by three washings with ice-cold Na^+^-free HBBS. Cells were lysed and mixed with Microscint20 (Perkin-Elmer Life Sciences). The radioactivity was measured with a scintillation counter (TopCount NXT, Perkin-Elmer Life Sciences).

### ^3^H-leucine efflux assay

The same protocol as for the uptake was used with the following differences after the initial washing- and starvation-step. Cells were preloaded for 5 min at 37 °C in the Na^+^-free HBBS containing 30 μM L-[^3^H]leucine (60 Ci/mmol). After washing three times with Na^+^-free HBBS (4 °C), efflux of radioactivity was induced by incubation in the presence or absence of indicated concentrations of test compounds for 1.5 min at 37 °C. The medium was then collected and its radioactivity was counted. The cells were washed three times with ice-cold Na^+^-free HBBS. Cells were lysed and mixed with Microscint20 (Perkin-Elmer Life Sciences). The radioactivity was measured with a scintillation counter (TopCount NXT, Perkin-Elmer Life Sciences). The L-[^3^H]leucine efflux values were expressed as percentage radioactivity (radioactivity of medium)/(radioactivity of the medium + radioactivity of the cells)).

### Statistical analysis

Data are expressed as average ± SD. Statistical analyses were conducted using GraphPad Prism 7 (GraphPad Software). IC_50_ and IG_50_ values were calculated by nonlinear regression. Proliferation assays at different amino acid concentrations were analyzed by multiple t-test with Holm-Sidak correction. Transcript levels of amino acid transporters in thyroid tumors were analyzed with a Kruskal-Wallis test with Dunn’s correction. Drug-induced *Slc7a5* reduction was analysed by an ordinary one-way ANOVA test. Tumor growth in vivo was analyzed with a two-way ANOVA test with Bonferroni correction. Proliferation and apoptosis markers of tumors were compared with a two-tailed Mann-Whitney test. Patient survival was analyzed with a log-rank test. Gene co-occurrence analysis was analyzed with a one-sided Fisher’s exact test. *P* ≤ 0.05 was considered as statistically significant; * = *p* < 0.05, ** = *p* < 0.01, *** = *p* < 0.001.

## Results

### Expression and functional characterization of LAT1 in thyroid cancer cell lines

**Fig. 1:**
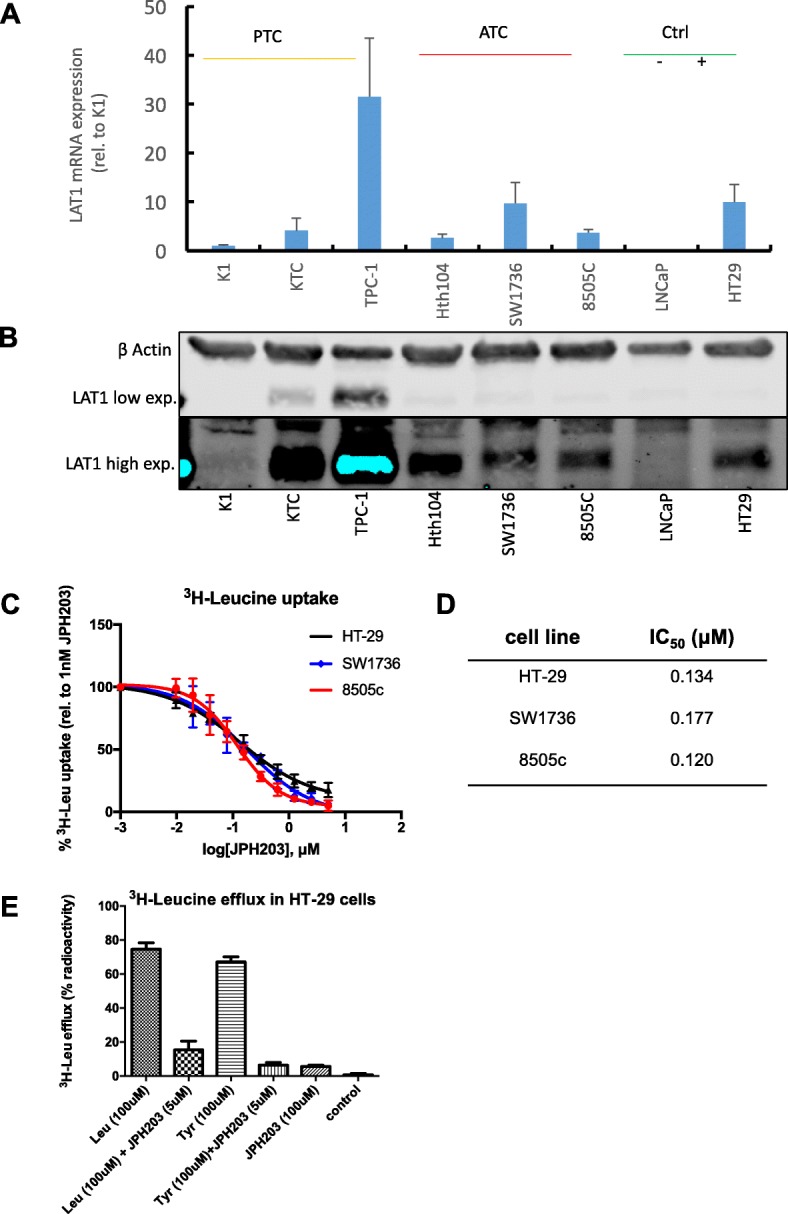
LAT1 is highly expressed in ATC cell lines and JPH203 inhibits leucine uptake. **a** *SLC7A5* transcription levels were quantified by Real-time PCR in three PTC cell lines (K1, KTC1, TPC-1) and three ATC cell lines (8505c, SW1736, Hth104). HT-29 cells served as positive control and LNCaP cells as negative control. LAT1 transcription levels were normalized to β-actin and plotted as fold change relative to K1 transcription level. **b**: LAT1 protein expression was quantified by western blotting in the cell lines described in (**a**). HT-29 cells served as positive control and LNCaP cells as negative control to confirm antibody specificity. Integrated signal of the LAT1 band was normalized to β-actin and displayed as fold change relative to K1. **c** ^3^H-Leucine uptake assay was performed in 8505c, SW1736, and HT-29. Dose-response curve was conducted in triplicate and ^3^H-Leucine uptake at each concentration was normalized to the uptake at the lowest JPH203 concentration (1 nM). These results represent the average of three independent experiments. **d** IC_50_ values for each cell line were calculated from **(c)**. **(e)**: Efflux of preloaded L-[^3^H]leucine induced by the two substrates leucine and tyrosine, and the inhibitor JPH203. The efflux of preloaded L-[^3^H]leucine was measured for 1.5 min in the presence or absence (*control*) of extracellularly applied substrates (100 μM leucine or tyrosine) and/or inhibitor (5 μM or 100 μM JPH203). The radioactivity released from the cells was expressed as percent of the total preloaded radioactivity (% radioactivity). Leucine efflux of the control (30–40% radioactivity over all experiments) was set as 0% radioactivity and the samples were normalized to the control

*SLC7A5* transcripts were found in all 6 thyroid cancer cell lines tested (Fig. 1a) with variable levels. In the HT-29 colon carcinoma cell line, *SLC7A5* transcription was 8-fold higher compared to K1 (used as basal level = 1), whereas in the prostate cancer cell line LNCaP, *SLC7A5* transcripts were undetectable (Fig. 1a).

We next evaluated LAT1 expression by western blotting. The specificity of the antibody employed was confirmed by the absence of signal in LNCaP cells and a strong signal in HT-29 cells (Fig. 1b). LAT1 expression was found in all cell lines with TPC-1 expression the highest level and K1 the lowest.

To explore the effects of blocking leucine transport through LAT1 in thyroid cancer cell lines we synthesized the highly specific LAT1 inhibitor JPH203 [31]. ^3^H-leucine uptake was measured for 8505c, SW1736 and HT-29 cells in the presence of different concentrations of JPH203 (Fig. 1c). HT-29 cells served as positive control. The IC_50_ values for each cell line are summarized in Fig. 1d. ^3^H-leucine uptake was not affected by JPH203 in LNCaP (Additional file 1: Figure. S1), in agreement with the lack of LAT1 expression in these cells. To better understand the mechanism of action of JPH203 we compared its effect on ^3^H-leucine efflux to L-tyrosine and L-leucine, which are both substrates [32]. As shown in Fig. 1e, JPH203 behaves as a non-substrate and possibly competitive inhibitor of LAT1, which prevents the efflux of ^3^H-leucine induced by leucine and tyrosine.

### JPH203 effect on cell proliferation is dependent on EAA concentration and LAT1 expression

**Fig. 2:**
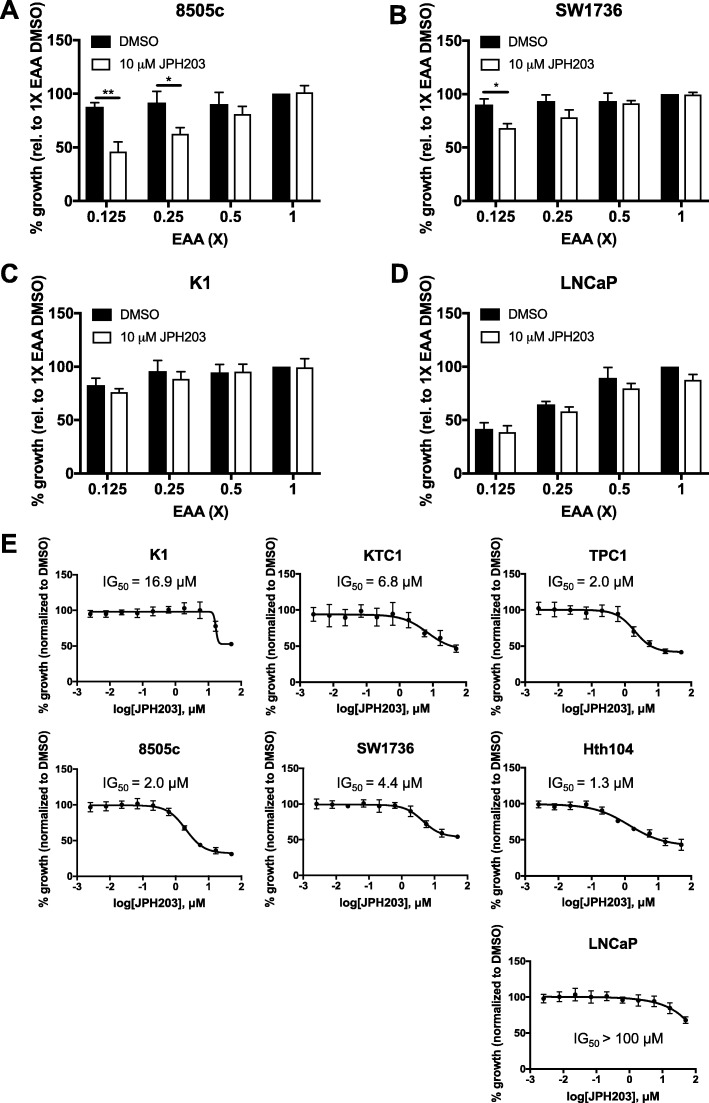
JPH203 reduces proliferation of the ATC cell lines 8505c and SW1736 when EAA concentrations in medium were lowered to plasma level. Proliferation assays of two ATC cell lines (**a**) 8505c and (**b**) SW1736, the PTC cell line (**c**) K1 and (**d**) LNCaP cells as negative control were performed at different EAA concentrations (1X - 0.125X, see Table S1 for detailed composition). Cells were plated in sixplicate using normal growth medium, after overnight incubation cells were washed with PBS and medium containing specific EAA concentration was added. Cells were treated (DMSO/10 μM JPH203) and fixed after (**a-c**) 48 h or (**d**) 96 h (two population doubling times). Each condition was normalized to the proliferation of DMSO-treated 1X EAA. These results represent the average of three independent experiments. Statistical analysis was performed by using multiple t-tests, one per row, and Holm-Sidak correction for multiple comparisons. **e** Dose-response curves for JPH203 were conducted with three PTC cell lines (K1, KTC1, TPC-1) and three ATC cell lines (8505c, SW1736, Hth104) and LNCaP cells as a negative control using medium containing 0.125X EAA (see Table S1 for detailed composition). Cells were plated in triplicate using normal growth medium, after overnight incubation cells were washed with PBS and 0.125X EAA was added. Cells were treated with different concentrations of JPH203 between 2.5 nM and 50 μM or DMSO as control. 8505c, SW1736, Hth104, KTC1, TPC-1 and K1 cells were incubated for 48 h; LNCaP cells for 96 h (two population doubling times) and growth was normalized to DMSO. These results represent the average of three independent experiments.

The concentration of most EAAs in conventional media (Table S1) greatly exceeds the level found in plasma (supplementary data of [33]). In order to mimic physiological leucine concentration we prepared a customized culture medium that contained various concentrations of EAA in fractions of the standard cell culture levels (1X EAA). JPH203 at 10 μM did not reduce cell proliferation at 1X EAA (Fig. 2) in any cell lines. However, when EAA concentrations were reaching plasma level (between 0.25/0.125X EAA), the proliferation of 8505c (Fig. 2a) and SW1736 cells (Fig. 2b) was significantly reduced by JPH203, while neither K1 nor LNCaP proliferation was significantly affected even at low concentrations of EAA (Fig. 2c and d ) by this 10 μM dose of JPH203. Dose-response inhibition curves of JPH203 were then performed in medium supplemented with 0.125X EAA. K1 cells were the least sensitive with a relative IG_50_ of 16.9 μM (Fig. 2e) while the three ATC cell lines and the PTC cell line TPC1 presented higher sensitivity to the drug with relative IG50 ranging between 1.3 and 4.4 μM. Furthermore, in LAT1-negative LNCaP cells the relative IG_50_ was extrapolated from the curve to be over 100 μM (Fig. 2e).

### LAT1 inhibition affects mTORC1 signaling

**Fig. 3:**
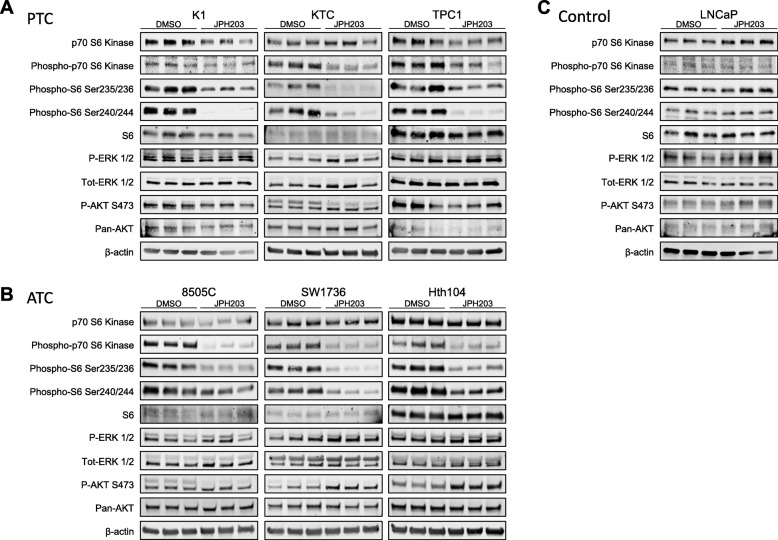
JPH203 decreases mTORC1 activity, but not ERK or AKT Western blot analyses of mTORC1, ERK and AKT activity in thyroid cancer cell lines after JPH203 treatment. P-p70 S6 kinase, p-ERK and p-AKT levels were assayed in (**a**) PTC cell lines and (**b**) ATC cell lines and (**c**) LNCaP as negative control. Cells were plated in triplicates using normal growth medium, after overnight incubation cells were washed and 0.125X EAA was added. Cells were treated with 10 μM JPH203 or DMSO as control and lysed after 24 h

Since LAT1 participates in mTORC1 activity [3] we measured Phospho-p70S6K and Phospho-S6 levels as a readout of mTORC1 in JPH203-treated cells. Both levels were significantly decreased in all thyroid cancer cell lines tested by 10 μM JPH203 when cultured in 0.125X EAA medium (Fig. 3a and b). In LNCaP cells, JPH203 did not alter p-S6 levels consistently under these conditions (Fig. 3c). The ERK pathway was not altered by this treatment in any cells. AKT phosphorylation was not reduced and sometimes further elevated (SW1736 in Fig 3).

### Blocking LAT1 exerts a strong cytostatic effect on thyroid cancer growth in vivo

**Fig. 4:**
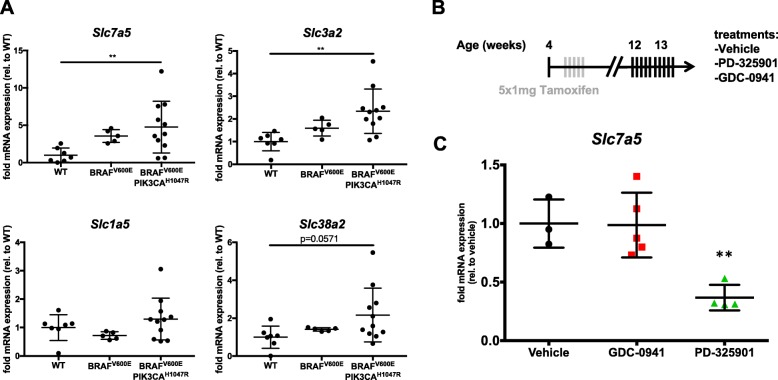
The inhibition of LAT1 by JPH203 induces a cytostatic growth arrest in an ATC mouse model in vivo. **a** *Slc7a5*, *Slc3a2*, *Slc1a5* and *Slc38a2* transcription levels were quantified in BRAF^V600E^ single-mutant (*n* = 5) and BRAF^V600E^/PIK3CA^H1047R^ double-mutant (*n* = 11) mouse thyroid tumors 4 months after induction and compared to wild-type thyroids (*n* = 7) level. Transcription levels of each gene were normalized to *Tuba1a* and plotted as fold change relative to wild-type level. Statistical analysis was performed using a Kruskal-Wallis test with Dunn’s correction for multiple comparisons. **b** Outline of experimental procedure, as described in the text. **c** *Slc7a5*, transcription level was quantified in BRAF^V600E^/PIK3CA^H1047R^ double-mutant mouse thyroid tumors after 10 days of treatments with PD-325901 at 5 mg/kg (*n* = 4) or GDC-0941 at 50 mg/kg (*n* = 5) or vehicle (*n* = 3)

Next, we investigated whether LAT1 was upregulated during the development of thyroid cancer in our mouse model [27]. Interestingly, *Slc7a5* and *Slc3a2* transcript levels were both significantly increased in BRAF^V600E^/PIK3CA^H1047R^ double-mutant tumors (4.8-fold and 2.3-fold, respectively) compared to wild-type samples. In BRAF^V600E^ single-mutant samples there was a tendency of increased *Slc7a5* and *Slc3a2* transcript levels, however this was not statistically significant (Fig. 4a). *Slc1a5* encoding ASCT2, which fuels the glutamine gradient necessary for LAT1 activity, was not found elevated in both single- and double-mutant tumors. Interestingly, the surrogate transporter SNAT2 encoded by *Slc38a2*, which is a sodium-dependent glutamine transporter, was 2.2-fold upregulated in BRAF^V600E^/PIK3CA^H1047R^ double-mutant tumors, which was close to being statistically significant (*p* = 0.0571, Fig. 4a). The mouse model was therefore shown to be suitable to explore if blocking LAT1 would affect thyroid cancer growth in vivo.

*Slc7a5* transcription has been shown to be driven by c-MYC [34]. We therefore tested if it was indeed the case in our model. BRAF^V600E^/PIK3CA^H1047R^ double-mutant Mice, two months after tumor induction, were treated with either a MEK inhibitor (PD-325901) and a class I Pi3’K inhibitor (GDC-0941) for ten days prior to dissection and LAT1 transcription analysis (Fig. 4b). While Pi3’K inhibition did not induced any change in *Slc7a5* transcript abundance, MEK inhibition induced more than 50% reduction.

**Fig. 5:**
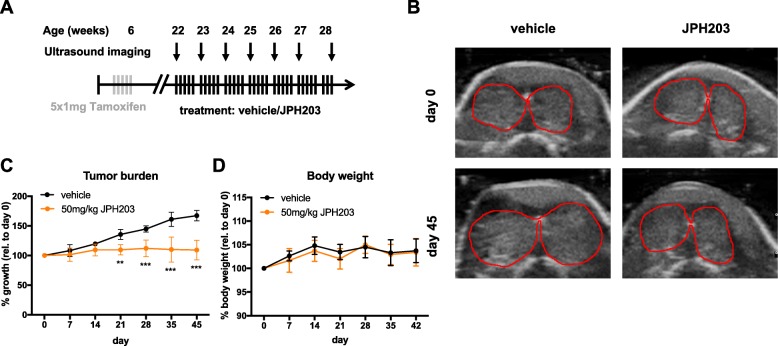
The inhibition of LAT1 by JPH203 induces a cytostatic growth arrest in an ATC mouse model in vivo. **a** Outline of experimental procedure, as described in the text. **b** Representative ultrasound images of vehicle- and JPH203-treated mice at day 0 and day 45. Images display the largest thyroid cross-section, marked with the red line, at each time point. **c** Quantification of the weekly acquired US images of vehicle- (*n* = 4) and JPH203-treated (n = 5) mice during the 45 days of treatment. Mice were treated for 5 consecutive days/week with either 50 mg/kg JPH203 or vehicle (Captisol®) by i.p. injection. The largest thyroid cross-section was analyzed once per week and normalized to the largest thyroid cross-section measured on day 0. Statistical analysis was performed using a two-way ANOVA test with Bonferroni correction for multiple comparisons. **d** Body weight was measured every week and normalized to the initial weight at day 0

Thyroid tumorigenesis was induced in 6-weeks old adult *Braf*^*CA/+*^*;Pik3ca*^*Lat/+*^*;Thyro::Cre*^*ERT2*^ mice by tamoxifen injection, which results in thyrocyte-specific expression of BRAF^V600E^ and PIK3CA^H1047R^. A cohort of 12 mice was separated into two groups that were treated with either vehicle or 50 mg/kg JPH203 i.p. daily for five days per week during 6.5 weeks (Fig. 5a). Weekly ultrasound imaging revealed that JPH203 exerted an arrest of tumor growth already after 14 days of treatment, whereas tumors of vehicle-treated mice continued to grow until the end of the experiment (Fig. 5c). Representative images comparing the tumor burden at day 45 (end of the study) to day 0 (start of treatment) illustrate the significant difference between vehicle- and JPH203-treated mice (Fig. 5b). In this model of thyroid cancer, all thyrocytes are undergoing recombination and transformation, inducing a cancerization of the whole thyroid. Therefore the assessment of whole thyroid size for tumor burden was used. Three mice (2 in the vehicle group, 1 in the JPH203 group) were excluded from the evaluation of the experiment, because they reached thyroid cancer-related end-point criteria (sudden > 15% bodyweight loss) before the end of the study. There was otherwise no difference in body weight between vehicle- and JPH203-treated mice (Fig. 5d). This suggests that JPH203 treatment has no striking adverse effects at the dosage employed (50 mg/kg i.p.).

### LAT1 inhibition increased TUNEL-positive nuclei and activated the amino acid stress response in thyroid tumors in vivo

**Fig. 6:**
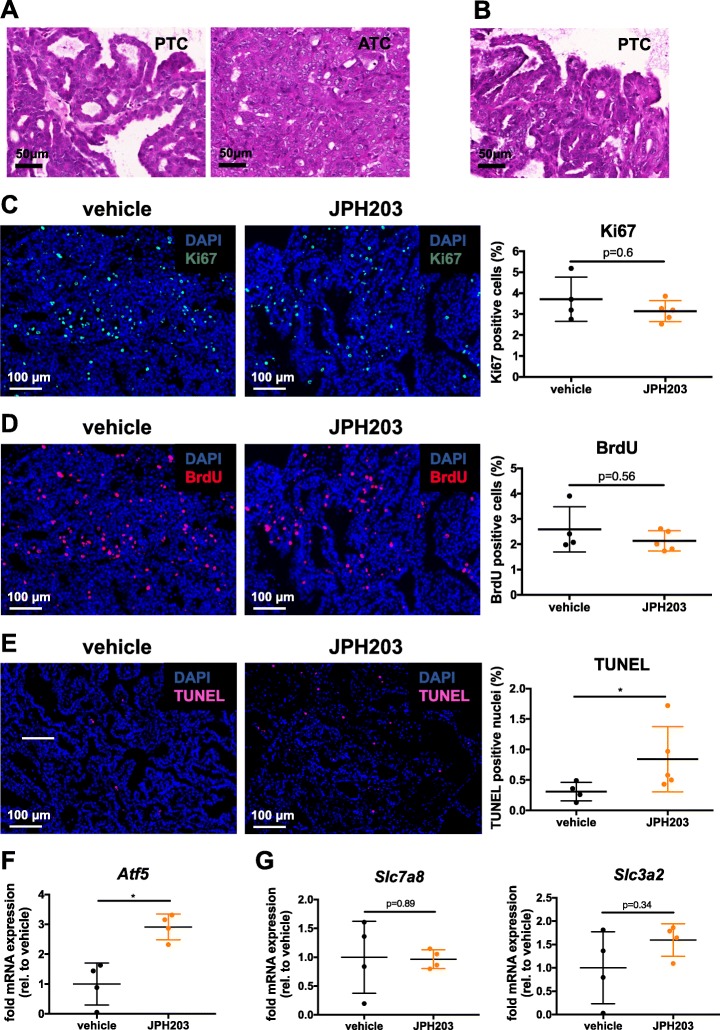
Tumors of JPH203-treated mice exhibit an increase in TUNEL-positive nuclei and activation of the amino acid stress response. H&E pictures demonstrating PTC/ATC features in (**a**) a vehicle-treated mouse and PTC features in (**b**) a JPH203-treated mouse. **c**, **d** Ki67-positive cells and BrdU-positive cells were quantified from each mouse and plotted in the graphs. **e** TUNEL-positive nuclei were quantified from each mouse and plotted in the graph. **f**, **g** *Atf5, Slc7a8 and Slc3a2* transcription levels were quantified in vehicle- and JPH203-treated animals. Transcription levels of each gene were normalized to *Tuba1a* and plotted as fold change relative to vehicle level. All statistical analyses were performed by using a two-tailed Mann-Whitney test

Hematoxylin/Eosin stains of thyroid sections revealed that 1 out 4 controls presented signs of progression from PTC to ATC (Fig. 6a) while none of the JPH203 treated animals presented ATC progression (Fig. 6b). We then analyzed tissue sections by immunofluorescence staining to quantify proliferation markers (Ki67, BrdU) and apoptosis marker TUNEL. The number of both Ki67- (Fig. 6c) and BrdU-positive cells (Fig. 6d) were reduced in JPH203-treated mice (15% and 17%, respectively), however, this decrease was not statistically significant. On the other hand, we found a statistically significant elevation of TUNEL-positive bodies in JPH203-treated tumors (*p* = 0.032) compared to vehicle-treated tumors (Fig. 6e). We then evaluated the JPH203 treatment efficiency in tumor cells in vivo. The transcription factor *ATF5* has been shown to be highly transcriptionally upregulated in vitro when cells are cultured in the absence of leucine [35] and is widely accepted as indicator of the amino acid stress response. Interestingly, *Atf5* transcript levels were significantly increased by 2.9-fold in JPH203-treated tumors (Fig. 6f). Finally we quantified the transcript levels of *Slc7a8* encoding LAT2, which is functionally equivalent to LAT1, and its heavy chain *Slc3a2*. Importantly, both *Slc7a8* and *Slc3a2* transcripts were not altered in JPH203-treated tumors (Fig. 6g).

### LAT1 expression is significantly increased in human ATC and associated with a shorter survival of patients diagnosed with PTC

**Fig. 7:**
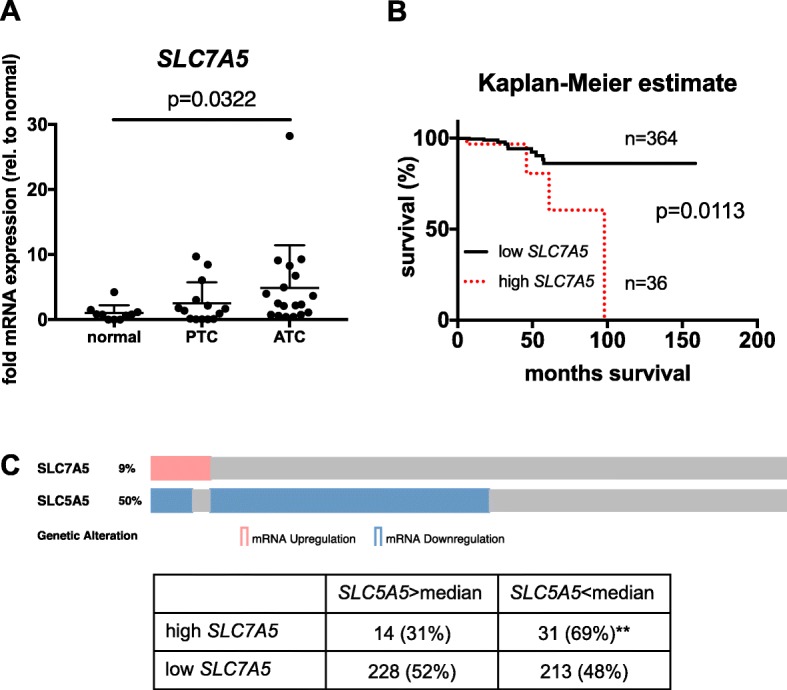
LAT1 is upregulated in human ATC and associated with shorter survival in PTC patients. **a** *SLC7A5* transcription levels were quantified in normal thyroid (*n* = 10), PTC (*n* = 14) and ATC (*n* = 18) samples derived from patients. Transcription levels of each gene were normalized to *ACTB* and plotted as fold change relative to normal thyroid. Statistical analysis was performed using a Kruskal-Wallis test with Dunn’s correction for multiple comparisons, *p*-value is indicated. **b** The PTC dataset (TCGA, Cell 2014) containing 400 tumor samples with RNAseq and survival data was analyzed using the cBioPortal online platform. Z-score > 1 was used to define samples with high *SLC7A5* expression (*n* = 36) and z-score < 1 was used to define samples with low *SLC7A5* expression (*n* = 364). Statistical analysis was performed by using a log-rank test, p-value is indicated. **c** *SLC5A5* expression levels were analyzed in the PTC dataset from (**b**) and co-occurrence of *SLC5A5* downregulation (defined as expression < median) with high *SLC7A5* (z-score > 1) was quantified. Statistical analysis was performed by using a one-sided Fisher’s exact test

To address the importance of LAT1 in human thyroid cancers, we obtained samples from patients, including non-cancerous thyroids, PTC and ATC. *SLC7A5* transcription was significantly increased (4.9-fold) in the ATC group compared to normal tissues (Fig. 6a, p = 0.03). Similar to the mouse model, *SLC7A5* transcription levels were intermediate in PTC samples (Fig. 7a).

To further investigate the potential role of LAT1 in patients, the TCGA PTC dataset containing 400 tumor samples with RNAseq and survival data was analyzed [36] using the cBioPortal online platform [37, 38]. A z-score threshold of 1 was used to define samples with increased *SLC7A5* transcription. Survival analysis by Kaplan-Meier estimate revealed that patients with high *SLC7A5* had a median survival of 98 months, whereas the median survival of patients with lower *SLC7A5* transcription was undefined (Fig. 6b, *p* = 0.0113). Finally, we addressed whether transcript levels of the sodium iodide symporter (NIS, *SLC5A5*) were lower in the high *SLC7A5* group, because low *SLC5A5* levels were associated with radioiodine resistance [39]. Interestingly, there was a significant co-occurrence of *SLC5A5* downregulation with high *SLC7A5* transcription (Fig. 7c, *p* = 0.006). Furthermore we analysed two cohorts from the GEO NCBI portal containing ATC samples compared to non-tumoral tissues and/or PTC tissues. In both cases ATC tissues presented significantly elevated *SLC7A5* transcription compared to normal tissues and PTC (Additional file 1: Figure S2).

## Discussion

The increased metabolism that is observed in tumors requires elevated uptake of nutrients such as EAAs. The EAA transporter LAT1 (*SLC7A5*) might play a crucial role in this process, as it shows increased transcription and/or expression in various tumors versus normal tissues [40]. However, the evidence of a causal relationship between LAT1 expression and tumor growth has been addressed only in few reports [31, 41]. Additionally, downregulation of *SLC7A5* by shRNAs inhibited growth of prostate [42] and gastric cancer cells [43], but did not affect proliferation of cholangiocarcinoma cells [44] or ovarian cancer cells [45]. Because of this apparent tissue specificity of LAT1 in tumors, we became interested in evaluating the role of this amino acid transporter in thyroid cancer in vitro and, more importantly, in vivo.

In experiments assessing the transcription levels of *SLC7A5* by TaqMan qPCR in different thyroid cancer cell lines the prostate cancer cell line LNCaP was used as a negative control [46] and the colon cancer cell line HT-29, in which LAT1 is highly and functionally expressed at the plasma membrane [31] served as positive control. The expression of LAT1 in all thyroid cancer cell lines was detectable at various levels (Fig. 1a and b).

In order to use a suitable pharmacological tool we synthesized the selective LAT1 inhibitor JPH203, which is currently the only potent LAT1-specific inhibitor available. ^3^H-leucine uptake was reduced by 90% at concentrations of 1 μM JPH203 in the two ATC cell lines that were analyzed, which demonstrated the dominant role of LAT1-dependent leucine uptake in these cells (Fig. 1c). Our control cell line HT-29 presented the expected potent inhibition of leucine uptake by JPH203 that was similar to the inhibition in 8505c and SW1736, while leucine uptake was not affected by JPH203 in LNCaP cells lacking LAT1 expression (Additional file 1: Figure S1). Because the compound did not induce efflux of ^3^H-leucine, which is typically observed by substrates like tyrosine or leucine [32], but efficiently inhibited this process we conclude that JPH203 is a non-substrate inhibitor that likely is competitive. This is also supported by the fact that proliferation inhibition by JPH203 was only observed when the EAA concentration was reduced (Figs. 2e). This is consistent with the recently published report showing that in a leukemia model, the JPH203 anti-proliferative effect is reduced by addition of EAA but not by NEAA [41].

We cannot exclude minor LAT1 independent leucine uptake, as seen in LAT1 deleted LS174T cells that continue to grow in DMEM medium [47], but at low EAA concentrations the ATC cell lines were almost solely depending on LAT1 for their leucine uptake.

Blocking LAT1 by JPH203 reduced proliferation of 5 out of 6 thyroid cancer cells with relative IG_50_ ranging from 1.3 μM to 6.8 μM. The PTC cell line K1 was less sensitive (16.9 μM), reflecting the reduced LAT1 expression in this line. In agreement with these findings, JPH203 at concentrations below 15 μM did not display any anti-proliferative effect on LNCaP cells with a relative IG_50_ calculated above 100 μM, while for HT-29 cells an IG_50_ of 4.1 μM was reported [31]. Thus, only at full blockage of leucine uptake an anti-proliferative effect was seen. Although we cannot exclude any unspecific effects of JPH203 in ATC cell lines, the fact that JPH203 did not affect proliferation of LNCaP cells below 10 μM indicates that off-target effects are unlikely at this concentration.

Nicklin et al. have well studied the relationship between LAT1-dependent leucine uptake and mTOR [3]. Here, we used Phospho-S6 and Phospho-p70S6K as readouts for mTORC1 activity and found a decrease of Phospho-S6 and Phospho-p70S6K levels in all JPH203-treated thyroid cancer cells which indicates that mTORC1 activity is affected (Fig. 3). Interestingly we found that the ERK pathways was not negatively affected by the drug, and neither was AKT phosphorylation. This demonstrates the specificity of the drug and that the effect observed is not a general deterioration of the condition of the cells. mTORC1 alteration is consistent with a previous report, which showed that LAT1 inhibition by JPH203 reduced mTORC1 activity in T-cell lymphoma cells [41]. Apart from leukemia cells, blocking all LATs (LAT1–4) by BCH reduced mTORC1 activity in HeLa cells [3]. However, to our knowledge, this is the first report indicating that the specific inhibition of LAT1 by JPH203 affects mTORC1 in carcinoma cells. This suggests that LAT1 could be used to modulate mTORC1 in a tumor-specific manner, potentially avoiding the side effects of systemic mTOR inhibition.

Noteworthy, at low levels of EAA (0.25× or 0.125×) the leucine concentration is closer to the physiological levels observed in plasma (Additional file 1: Table S1 and supplementary material of [33]. In order to translate the above observations to an in vivo situation we took advantage of a genetically engineered mouse model of thyroid cancer. In this model, which thyroid-specifically express BRAF^V600E^ and PIK3CA^H1047R^ mutations leading to constitutively active MAPK and Pi3’K pathways, it was previously demonstrated that 3–6 months after tamoxifen induction the tumors display both PTC and ATC features [27]. Based on this, we selected the four-months time point to start the JPH203 treatment to subsequently asses its effect on thyroid growth in late-stage disease. *Slc7a5* transcription was increasing with tumor progression from wild-type to BRAF^V600E^ single-mutant tumors, which correspond to benign and PTC lesions, and BRAF^V600E^/PIK3CA^H1047R^ double-mutant tumors that consisted of PTC and ATC foci (Fig. 4a). Concomitantly, the transcription level of *SLC7A5* was lower in the PTC cell line K1 when compared to the ATC cell lines 8505c and SW1736 (Fig. 1a). This is consistent with the fact that LAT1 is associated with invasive behavior of cancer cells [48, 49] and that ATC is a very invasive highly aggressive tumor subtype in which most patients are not suitable for surgery anymore due to local invasion at initial diagnosis [19]. Similarly to studies indicating that 4F2hc (*SLC3A2*) is overexpressed during the progression of breast and pancreatic cancer [8, 11], we detected significantly increased transcript levels of *Slc3a2* in BRAF^V600E^/PIK3CA^H1047R^ double-mutant tumors (Fig. 4a). Our data indicate that 4F2hc expression increased in parallel with LAT1 in order to enhance LAT1 activity in thyroid cancer. Finally, we found that ASCT2 (*Slc1a5*), which works together with LAT1, was not altered in BRAF^V600E^ single-mutant tumors or BRAF^V600E^/PIK3CA^H1047R^ double-mutant tumors. However, in the BRAF^V600E^/PIK3CA^H1047R^ double-mutant tumors the functionally equivalent transporter *Slc38a2* (SNAT2) was 2.2-fold increased, although not statistically significant it could still partially compensate the lack of increased *Slc1a5* transcription (Fig. 4a). Thus, in this mouse model all the partners required for LAT1-dependent leucine uptake were transcribed and in some cases upregulated with tumor progression. Since c-MYC has been shown in the context of pancreatic cancer cells to drive *SLC7A5* transcription [34], in our mouse model we have tested the relative role of the RAF-MEK-ERK and Pi3’K pathways for *Slc7a5* transcription. MEK inhibition was very potent in reducing *Slc7a5* transcripts while Pi3’K inhibition was not (Fig. 4b). This reflects the apparent predominant role of BRAF^V600E^ to induce LAT1 elevation. It was also worth noticing that while LNCaP cells were affected by EAA reduction, 8505c expressing LAT1 cells showed a resistance to this “amino acids starvation” (Fig. 2a and d). Importantly, JPH203 exposure restored “amino acids starvation” sensitivity in 8505c similarly to LNCaP cells. Furthermore amino acids concentration in blood and therefore in tissues are at similar levels as in our “amino acids starvation” condition (Additional file 1: Table S1). Taken together these date suggest that BRAF^V600E^ induces LAT1 elevations that confers tumor cells the ability to grow in the nutrient-limited environment.

To study the effect of LAT1 inhibition in thyroid cancer in vivo, mice were treated with a previously established effective dose of JPH203 at 50 mg/kg [41]. Oda et al. demonstrated that applying a dose of 25 mg/kg JPH203 in mice results in a plasma concentration of 2.4 μM that is maintained during at least 4 h [31]. Therefore, we assumed that the 50 mg/kg dose could result in a plasma concentration, sufficient to block LAT1 according to our in vitro data (Fig. 1 and 2). Interestingly, treated-mice presented a cytostatic effect with a tumor growth arrest after 14 days of treatment and subsequent stable disease (Fig. 5c). Proliferation markers (Ki67, BrdU) were not significantly decreased in JPH203-treated tumors (Fig. 6c and d) while apoptosis was elevated as demonstrated by TUNEL staining (Fig. 6e). This observation is compatible with mTOR inhibition that is often associated with increased apoptosis. The increase of apoptosis associated with no measurable modifications of tumor proliferation resulted in an apparent tumor size stabilization or “zero net gain”. Importantly, the strong 2.9-fold increase of *Atf5* transcription in JPH203-treated tumors suggests that LAT1 inhibition depleted EAA concentration in tumor cells in vivo (Fig. 6f). The fact that *Slc7a8*, which is functionally equivalent to LAT1, was not transcriptionally upregulated in JPH203-treated tumors indicates that this transporter was not able to compensate for the lower LAT1 activity (Fig. 6g). For the most aggressive cases with a survival median of 5 months such effect would most likely translate into clinical benefit.

The increased *Slc7a5* transcription found in the mouse model (Fig. 4a) is consistent with the findings in human ATC (Fig. 7a) underscoring that LAT1 could also be involved in human ATC tumors. In addition, in the dataset available at TCGA Data Portal, focusing on PTC [36] showed a significant difference in the 5-year survival between patients with high and low *SLC7A5* levels. The estimated long-term survival was significantly reduced for patients with high *SLC7A5* transcript levels (Fig. 7b) even if the disease progression free analysis did not show any difference (data not shown). While these data have to be taken carefully, further analyses from GEO datasets [50, 51] showed SLC7A5 increase from normal tissue to PTC and ATC (Additional file 1: Figure. S2) emphasizing the fact that SLC7A5 is associated with the most aggressive untreatable cases of thyroid cancers. Finally, the finding of high *SLC7A5* levels significantly co-occurring with low *SLC5A5* transcripts (Fig. 7c) might indicate that LAT1 is associated with aggressive forms of thyroid cancer like RAI refractory PTC. This is in line with reports on prostate cancer, pancreatic cancer and neuroendocrine lung tumors, in which high LAT1 expression was correlated with poor survival [7, 10, 11].

## Conclusions

Our data show that LAT1 plays a critical role in tumor growth in mice and mTORC1 activity in human thyroid cancer cell lines, suggesting that LAT1 helps cells grow in the physiological environment that is low in amino acids. Additionally, using a genetically engineered mouse model that recapitulates the development of human ATC, we observed significant growth arrest when LAT1 was inhibited by the non-substrate LAT1 inhibitor JPH203. This is the first study to show the preclinical benefit of blocking LAT1 for solid tumor in immunocompetent mice. Finally, in human thyroid cancer patients, LAT1 expression levels seem to correlate with tumor progression, poorer outcome and radioiodine resistance. Therefore, LAT1 should be considered as a potential therapeutic target in the development of new anticancer drugs in the context of unresectable and non iodine-treatable thyroid cancers.

## Additional file

Additional file 1: **Figure S1.** Leucine uptake in negative control cells LNCaP. **Figure S2.** LAT1 transcription levels in GSE datasets 65144 and 33630. **Table S1.** Comparison of the amino acid concentrations (in μM) in different media versus plasma. (PDF 104 kb)

## Abbreviations

ATC: Anaplastic thyroid carcinoma
EAA: Essential amino acids
LAT1: L-type amino acid transporter 1
NIS: Sodium iodide symporter
PTC: Papillary thyroid carcinoma
RAI: Radioactive iodine
