# Supplementary material for: The LAT1 inhibitor JPH203 reduces growth of thyroid carcinoma in a fully immunocompetent mouse model
Source: J Exp Clin Cancer Res. 2018 Sep 21;37:234. doi: 10.1186/s13046-018-0907-z (PMC6150977; doi:10.1186/s13046-018-0907-z)
Supplement: Supplementary file 1 — Figure S1. Leucine uptake in negative control cells LNCaP. Figure S2. LAT1 transcription levels in GSE datasets 65144 and 33630. Table S1. Comparison of the amino acid concentrations (in μM) in different media versus plasma. (PDF 104 kb) [file 13046_2018_907_MOESM1_ESM.pdf]

Supplementary informations, Häfliger *et al.*
(3 pages)

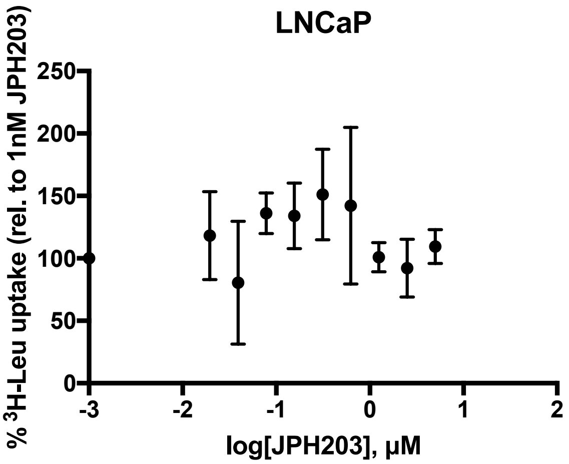

Figure S1: Leucine uptake in negative control cells LNCaP.

^{3}H-Leucine uptake assay was performed in LNCaP cells. Dose-response curve was conducted in triplicate and ^{3}H-Leucine uptake at each concentration was normalized to the uptake at the lowest JPH203 concentration (1 nM). These results represent the average of three independent experiments.

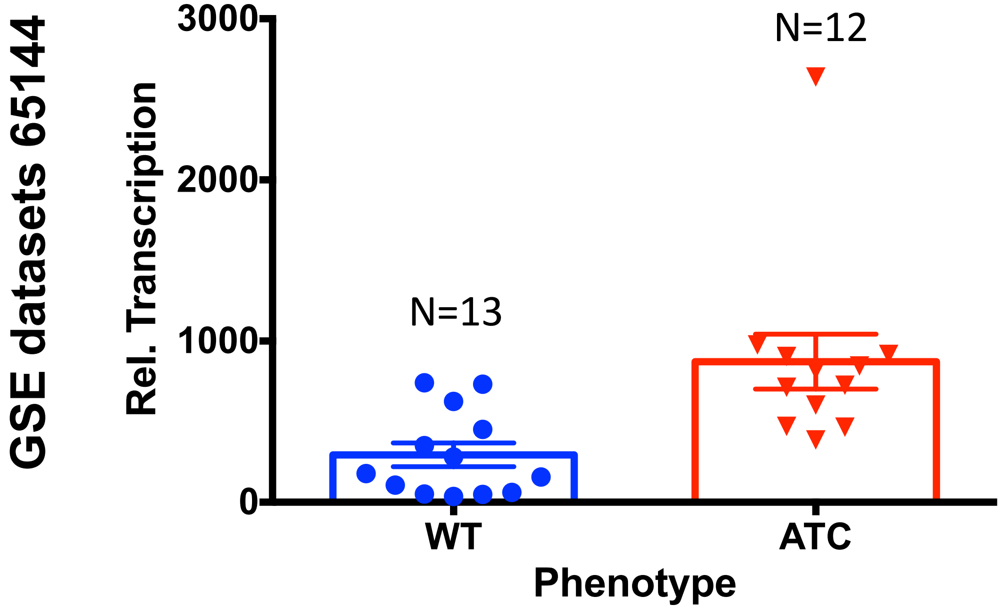

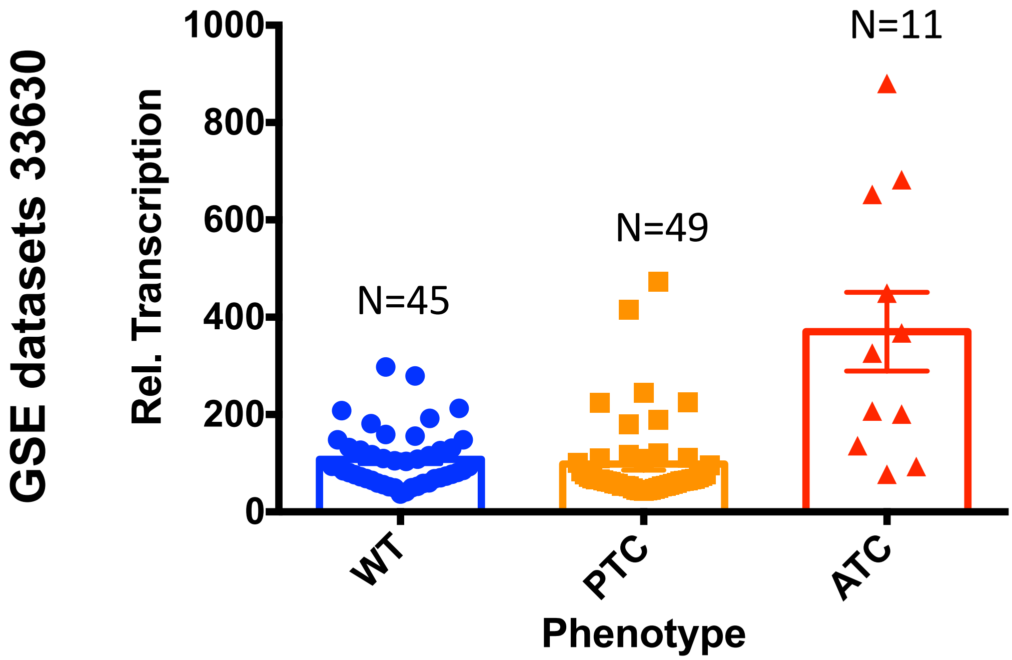

Figure S2: LAT1 transcription levels in GSE datasets 65144 and 33630.

Relative transcription (+/- SEM) in the GEO dataset GSE65144 (von Roemeling CA J Clin Metab 2015) and GSE33630 (Tomas-G Oncogene 2012) for LAT1/SLC7A5 (201195_s_at). p values for the upper panel is 0.006977 wit an unpaired t-test 2-tails assuming no equal SD (Welch's correction) and 1.019e-10 with a one-way annova test for the lower panel.

Supplementary Materials and methods

Analytical data JPH203 hydrochloride:

^{1}H NMR (400 MHz, DMSO-d_6) δ = 8.13 – 8.07 (m, 2H), 7.62 – 7.57 (m, 3H), 7.42 (s, 2H), 6.90 (d, J = 2.1 Hz, 1H), 6.85 (d, J = 2.0 Hz, 1H), 5.19 (s, 2H), 3.46 – 3.43 (m, 1H), 3.08 (dd, J = 14.3, 4.6 Hz, 1H), 2.87 (dd, J = 14.3, 7.8 Hz, 1H).
^{13}C NMR (101 MHz, DMSO-d_6) δ = 169.0, 161.9, 148.7, 146.7, 142.7, 141.3, 136.4, 131.6, 130.3, 129.3, 128.3, 126.9, 119.1, 114.0, 103.0, 69.6, 55.0, 35.7.

Amino acid	RPMI	1X MEM amino acids	0.125X MEM amino acids	Plasma*
L-Arginine	1148	599.1	74.9	98.5
L-Cystine	371.2	100	12.5	?
L-Histidine	96.7	200	25	81.6
L-Isoleucine	381.2	400	50	66.9
L-Leucine	381.2	400	50	128.6
L-Lysine	219	396.2	49.5	195.3
L-Methionine	100.5	101.3	12.7	27.3
L-Phenylalanine	90.8	200	25	62.2
L-Threonine	167.9	400	50	122.6
L-Tryptophan	24.5	50	6.25	58.9
L-Tyrosine	110.4	198.9	24.9	68.2
L-Valine	170.8	400	50	235.1

Table S1: Comparison of the amino acid concentrations (in µM) in different media versus plasma

Note: LAT1 substrates are shown in black

* Miyagi Y, Higashiyama M, Gochi A, Akaike M, Ishikawa T, Miura T, et al. Plasma free amino acid profiling of five types of cancer patients and its application for early detection. PLoS One. 2011;6:e24143.
